# The Effect of Adjustment of Endotracheal Tube Cuff Pressure during Scarless Remote Access Endoscopic and Robotic Thyroidectomy on Laryngo-Pharyngeal Complications: Prospective Randomized and Controlled Trial

**DOI:** 10.3390/jcm8111787

**Published:** 2019-10-25

**Authors:** Chang-Hoon Koo, Hye-Min Sohn, Eun-Su Choi, June-Yong Choi, Ah-Young Oh, Young-Tae Jeon, Jung-Hee Ryu

**Affiliations:** 1 Department of Anesthesiology & Pain Medicine, Seoul National University Bundang Hospital, Seongnam 13620, Korea; vollock9@gmail.com (C.-H.K.); ohahyoung@hanmail.net (A.-Y.O.); jeonyt@snubh.org (Y.-T.J.); 2 Department of Anesthesiology & Pain Medicine, Ajou University School of Medicine, Suwon 16499, Korea; sophiesohn21@gmail.com; 3 Department of Anesthesiology & Pain Medicine, Nowon Eulji Medical Center, Eulji University, Seoul 01830, Korea; potterydoll@gmail.com; 4 Department of Surgery, Seoul National University Bundang Hostpial, Seongnam 13620, Korea; aznagran@gmail.com; 5 Department of Anesthesiology & Pain Medicine, Seoul National University College of Medicine, Seoul 03080, Korea

**Keywords:** endotracheal intubation, postoperative complications, sore throat, thyroidectomy

## Abstract

Scarless remote access endoscopic and robotic thyroidectomy has been recently performed as a safe and feasible method. However, little is known about the laryngo-pharyngeal complications after surgery and the effect of adjusting the endotracheal tube cuff pressure during surgery on laryngo-pharyngeal complications. Patients were randomized into two groups: the control group (*n* = 52) and adjusted group (*n* = 52). The initial cuff pressure was set to 25 mmHg and then monitored without adjustment (control group) or with adjustment at approximately 25 mmHg (adjusted group) throughout surgery. The incidences and severity of postoperative sore throat (POST), hoarseness, dysphagia, and cough were recorded at 1, 6, 24, and 48 h after surgery. Cuff pressures of the control group changed significantly over time and were higher than those of the adjusted group. The incidence of POST was lower in the adjusted group at 24 h postoperatively (*p* = 0.035), and there was a significant difference in the severity of POST at 6 and 24 h postoperatively between the two groups. There were no differences in the incidence of hoarseness, dysphagia, and cough between the two groups, except dysphagia and cough at 6 h postoperatively. Therefore, intraoperative monitoring and adjustment of the cuff pressure can reduce the incidence of laryngo-pharyngeal complications.

## 1. Introduction

Minimally invasive techniques have been frequently used for thyroidectomy since their remote approach provides cosmetic benefit [1,2]. Previous reviews with a meta-analysis of minimally invasive video-assisted thyroidectomy suggested that minimally invasive thyroidectomy has similar perioperative complications and oncologic outcomes compared with conventional open thyroidectomy, and operative time was significantly longer in minimally invasive thyroidectomy than in conventional open thyroidectomy [1,2]. 

After thyroidectomy, laryngo-pharyngeal complications, including postoperative sore throat (POST), hoarseness, dysphagia, and cough, are common [3]. These complications can lead to distress, degrading the quality of life of patients during recovery and, therefore, the prevention and evaluation of these symptoms are required to enhance the quality of recovery and patient satisfaction. The incidence of POST was reported to be 65%–84% after thyroidectomy [4]. POST usually resolves spontaneously within a few days [5], but there are cases that persist long after the operation [3]. Scarless remote access endoscopic and robotic thyroidectomy (SERT) has been developed recently to minimize neck incisions and to offer great cosmetic satisfaction, but little is known about the laryngo-pharyngeal complications after SERT.

Many surgical and anesthetic factors contribute to POST after thyroidectomy [3,4]. Among the anesthetic factors, endotracheal intubation and endotracheal cuff pressure during surgery are reported to cause laryngeal injury and influence the degree of POST [5,6]. Manipulation of the thyroid, which is adjacent to the trachea, during thyroidectomy may cause variation in cuff pressure, which may increase the incidence and severity of POST after thyroidectomy [4,7]. Therefore, the adjustment of endotracheal tube cuff pressure during thyroidectomy has been investigated to decrease the incidence and degree of POST [8]. SERT has a relatively longer operative time compared with an open thyroidectomy. CO_2_ gas insufflation under the skin flap at certain pressures is needed, and the pressure at the working space may influence the endotracheal cuff pressure and laryngo-pharyngeal complications. However, little information is known about the effect of the adjustment of the endotracheal cuff pressure on the incidence and degree of POST during SERT.

It was hypothesized that monitoring and adjustment of the cuff pressure during SERT could reduce the incidence and degree of POST after surgery. This prospective, randomized, and controlled single-blinded trial evaluated the effect of intraoperative adjustment of the endotracheal tube cuff pressure during SERT on the incidence of laryngo-pharyngeal complications, including POST, hoarseness, dysphagia, and cough. 

## 2. Materials and Methods

### 2.1. Study

The protocol of this prospective, randomized, and single-blind trial was approved by the Institutional Review Board of Seoul National University Bundang Hospital (IRB No. B-1606-351-006) and registered in the Clinical Research information Service (CRiS) (registration No. KCT0002365). Written informed consent was received from all patients after explanation of the study during the preoperative visit. 

### 2.2. Patients

A total of 104 patients aged 19–70 years with an American Society of Anesthesiologists (ASA) physical status of I–II and who were scheduled to undergo elective SERT between June 2016 and November 2017 were included. After preoperative vocal cord examination, patients with freely movable vocal cords were included in this study. 

Anesthetic exclusion criteria were preoperative sore throat, hoarseness, dysphagia, asthma, gastroesophageal reflux, obesity (BMI > 30 kg/m^2^), symptoms of upper respiratory infection within 2 weeks of surgery, difficult airway (requiring more than two attempts or more than 15s for endotracheal intubation), and previous surgeries of the oral cavity or pharynx. Surgical inclusion criterion was intraparenchymal thyroid cancer with a size of less than 2 cm. Thyroid cancer with goiter or extrathyroial extension and cases without definitive identification of recurrent laryngeal nerve, or with mechanical or thermal injury of recurrent laryngeal nerve during surgery, were excluded.

### 2.3. Anesthesia

Patients were premedicated with intravenous midazolam 0.03 mg/kg at the reception area of the operating room. In the operating room, standard monitoring was done with electrocardiography, noninvasive blood pressure measurement, and pulse oximetry. In addition, the depth of the neuromuscular blockade was monitored using acceleromyography (TOF-Watch-SX, Organon Ireland Ltd., a subsidiary of Merck & Co., Inc., Swords, Co. Dublin, Ireland) at the adductor pollicis muscle.

Total intravenous anesthesia with propofol and remifentanil was used for induction and maintenance of anesthesia using target controlled infusions. After loss of consciousness, TOF-Watch was calibrated and measured every 15 seconds during the induction of anesthesia. Rocuronium 0.6 mg/kg was administered and additional doses of rocuronium 0.15 mg/kg were used, if needed, to reach one to two twitches on train-of-four stimulation of the ulnar nerve. During intubation, the type of blade was standardized with a Macintosh laryngoscope, and appropriately sized electromyogram (EMG) reinforced tubes were used to monitor recurrent laryngeal nerve intraoperatively. Subsequently, patients were ventilated with oxygen and air to maintain an end-tidal CO_2_ of 35–40 mmHg. The depth of anesthesia was monitored and maintained using a bispectral index between 40 and 60 during the operation.

After surgery, residual neuromuscular blockade was reversed with neostigmine 0.04 mg/kg and glycopyrrolate 0.01 mg/kg. Confirming spontaneous breathing and consciousness, patients were transferred to the postanesthesia care unit (PACU) after extubation.

### 2.4. Surgery

All SERT was performed by a single experienced surgeon to keep a constant surgical stimulus, as previously described [9]. Patients were in supine position, and a pillow was placed under the shoulder to extend the neck. Skin flaps were marked, and 200 mL of diluted epinephrine solution was injected subcutaneously over the subplatysmal space and upper chest area. Bilateral circumareolar incisions were made at the superomedial margin of each areolar, with two 8 mm axillary incisions. The flap was dissected and extended to the thyroid cartilage superiorly, 3 cm below the clavicle inferiorly, and laterally, from just beyond the lateral border of one sternocleidomastoid muscle to the other. Gas insufflation to the working space was done with CO_2_ gas at a pressure of 5–6 mmHg through the 12 mm port. Then, robotic devices were docked, and the midline was separated by a monopolar electrocautery until the thyroid was visible. The isthmus was separated using an ultrasonic shear after the identification of the cricothyroid membrane, isthmus, and central group of lymph nodes. The middle thyroid pedicle was ligated and divided by a harmonic shear, and the thyroidectomy was performed, which confirmed the middle and inferior thyroid pedicles, recurrent laryngeal nerve, as well as the superior and inferior parathyroid gland. A unilateral thyroid specimen was removed through the left axillary incision via an endopouch, and the contralateral lobe was dissected in the same manner. Anti-adhesive solution was applied on the whole flap after thyroidectomy, and one Jackson-Pratt drain was placed through the left axillary incision. A surgical brassiere was applied to compress the flap after surgery.

### 2.5. Randomization

Patients were randomized before the induction of anesthesia by an independent anesthesiologist in charge of patient allocation. A computer-generated random number table (random allocation software v 1.0, Isfahan University of Medical Sciences, Isfahan, Iran) with block size 4 randomly assigned patients to either the control (*n* = 52) or adjusted (*n* = 52) group. Patients and outcome assessors were blinded to the group assignment, whereas the anesthesiologist in charge of patient allocation during anesthesia was not blinded to group assignment.

### 2.6. Intervention

Intervention according to group assignment was as follows:

- Control group: After endotracheal intubation, the cuff pressure of the endotracheal tube was adjusted to 25 cm H_2_O with a manometer right after intubation. The cuff pressure was monitored, but was not adjusted during the thyroidectomy, except to maintain the cuff pressure within the range of 20–50 cm H_2_O to avoid pulmonary aspiration or tracheal mucosal injury throughout the operation [10,11].
- Adjusted group: The endotracheal cuff was initially inflated and adjusted to achieve a cuff pressure of approximately 25 cm H_2_O. The cuff pressure was monitored continuously and maintained at 25 cm H_2_O with a manometer throughout thyroidectomy.

**Figure 1:**
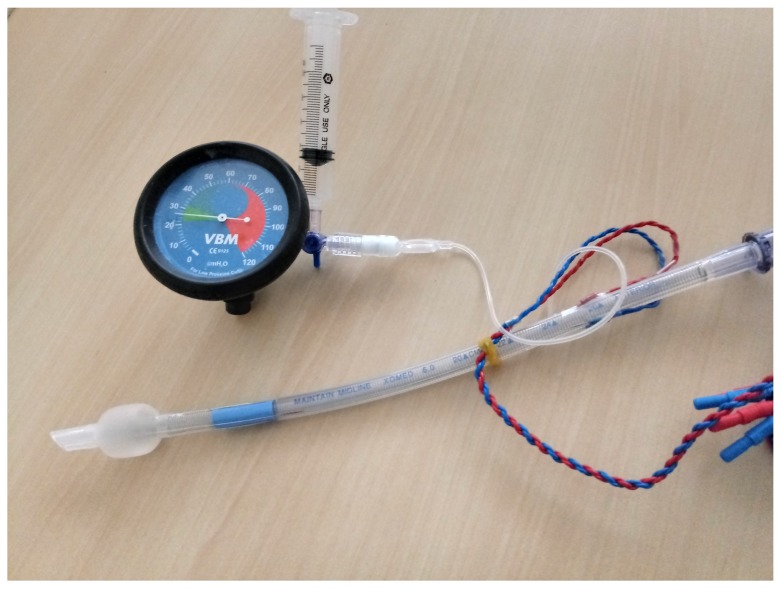
Cuff pressure measurement system. The cuff pressure was continuously monitored using a manometer. The pilot balloon of the endotracheal tube was connected to the manometer via a 3-way stopcock. Air was injected into or withdrawn from the pilot balloon using a 10-mL syringe via a side port on the stopcock.

The cuff pressure was monitored continuously as follows:

- The pilot balloon of the endotracheal tube was connected to manometer via a 3-way stopcock. Air was injected into or withdrawn from the pilot balloon using a 10-mL syringe via a side port on the stopcock (Figure 1).

### 2.7. Assessment of Outcomes

The primary outcome was the incidence of POST. Secondary outcomes included the severity of POST, incidence of hoarseness, dysphagia, and cough. Laryngo-pharyngeal symptoms, including POST, hoarseness, dysphagia, and cough, were evaluated at 1, 6, 24, and 48 h after surgery by one outcome assessor who was blinded to group assignment. Outcomes were defined according to a previous study [8]. POST was defined as pain at the larynx or pharynx; dysphagia was defined as difficulty in swallowing; hoarseness was defined as a harsh or strained voice; cough was defined as a sudden reflex that forces air out of the throat. The degree of POST was evaluated with 4 degrees [12]—None: No POST; mild: POST when swallowing; moderate: POST during rest that worsens when swallowing; and severe: severe POST requiring analgesics. Patients were evaluated using the modified Aldrete scoring system until ready for discharge from the PACU, and the criterion used for patient discharge from PACU was the achievement of a modified Aldrete score of 9 [13]. All patients were instructed to evaluate their symptoms on a numerical rating scale (NRS, on a scale from 0, no pain, to 100, most severe pain imaginable) during the preoperative visit, and postoperative pain were evaluated using the NRS at postoperative 1, 6, 24, and 48 h after surgery. The amount of drainage was recorded at postoperative 6, 24, and 48 h. 

### 2.8. Sample Size Calculation

A sample size of 104 was calculated based on the result of the previous investigation [8]. The incidence of POST at 24 h after thyroidectomy was 66% for the control group [8], and a decrease of the incidence of sore throat from 66% to 36% was considered to be clinically significant. According to the power analysis program (G * power 3.1), 43 patients were necessary per group for a power of 80% and α error of 0.05. To account for a dropout rate of 20%, 52 patients were included in each group.

### 2.9. Statistical Analysis

SPSS 22.0 for Windows (SPSS, Chicago, IL, USA) was used for data analysis. Incidence variables (gender, ASA physical class, operative extent, pathologic diagnosis, incidence and degree of POST, hoarseness, dysphasia, and cough) were analyzed using the χ2 test. Continuous variables (age, weight, height, anesthesia time, operation time, tumor size, resected thyroid weight, endotracheal cuff pressure, PACU stay, postoperative pain, and amount of drainage) were compared using the t-test. Data are presented as the number of patients (%) or as the mean (SD), unless stated otherwise. A full analysis set was used for data analysis. *p*-Values of <0.05 were considered statistically significant.

## 3. Results

### 3.1. Patients

**Figure 2:**
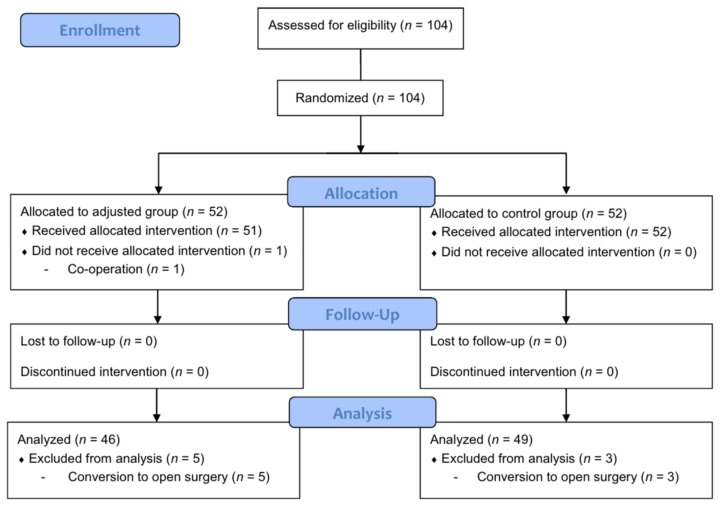
CONSORT diagram for the trial. One hundred four patients were randomized and 9 patients were excluded from the final analysis. Finally, a total of 95 patients (46 in the adjusted group, and 49 in the control group) were analyzed.

One hundred and four patients who underwent SERT between September 2016 and October 2017 at Seoul National University Bundang Hospital were enrolled. Eight patients (5 in the adjusted group, 3 in the control group) were excluded because of conversion to open surgery after the induction of anesthesia. One patient in the adjusted group was excluded because co-operation (ovarian cystectomy with thyroidectomy) was decided right before the induction of anesthesia. Therefore, a total of 95 patients were included in the final analysis (Figure 2). Patients and surgery characteristics are presented in Table 1. In addition, all patients were intubated on the first attempt, and the Cormack-Lehane classification of each patient was comparable between the two groups (*p* = 0.110).

### 3.2. Cuff Pressure of Endotracheal Tube

**Figure 3:**
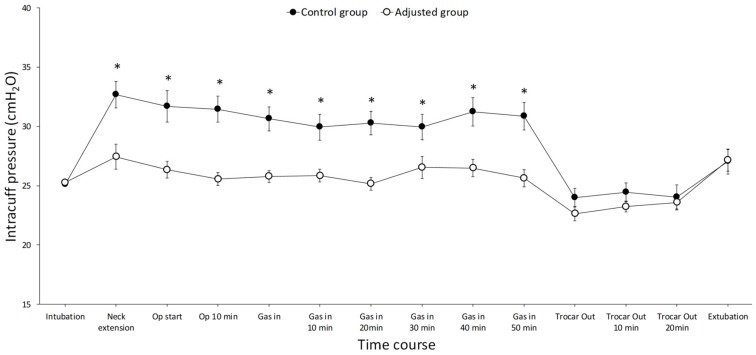
Changes in the cuff pressure during scarless remote access endoscopic and robotic thyroidectomy. There was a significant difference in the cuff pressure over time between the two groups; the cuff pressure was lower in the adjusted group than in the control group (*p* < 0.001). The intragroup analysis showed that there was a significant change in the cuff pressure in the control group during surgery (*p* < 0.001). Results are expressed as mean (SE).

No difference was observed in the baseline endotracheal cuff pressure between the two groups. However, repeated-measures ANOVA revealed that there was a significant difference in cuff pressure over time (*p* < 0.001); the cuff pressures in the control group were higher than those in the adjusted group. Additionally, there was a significant change in cuff pressure in the control group over time during anesthesia (*p* < 0.001) (Figure 3). The mean cuff pressure during surgery was 28.3 (interquartile range (IQR) 25.5–31.7) cmH_2_O in the control group and 25.0 (IQR 24.8–26.2) cmH_2_O in the adjusted group (*p* < 0.001).

### 3.3. Laryngo-Pharyngeal Complications

**Figure 4:**
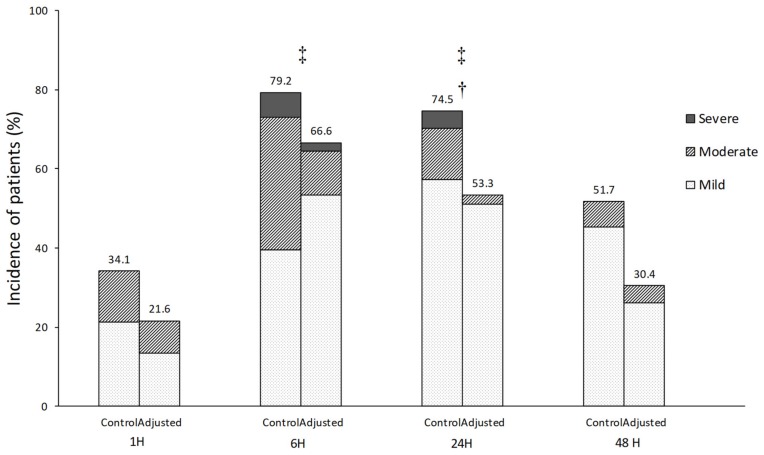
The incidence and severity of postoperative sore throat (POST). The incidence of POST was lower in the adjusted group than in the control group at 24 h postoperatively (53.3% vs. 74.5%, *p* = 0.035). The severity of POST was less severe in the adjusted group than in the control group at 6 h (*p* = 0.034) and 24 h postoperatively (*p* = 0.034). † *p* < 0.05, the incidence of POST; ‡ *p* < 0.05, the severity of POST.

There was a significant difference in the incidence and degree of POST between the two groups. The incidence of POST was lower in the adjusted group than in the control group at 24 h postoperatively (53.3% vs. 74.5%, *p* = 0.035) (Figure 4). Moreover, POST in the adjusted group was less severe than that in the control group at 6 h (*p* = 0.034) and 24 h postoperatively (*p* = 0.034) (Figure 4). 

**Figure 5:**
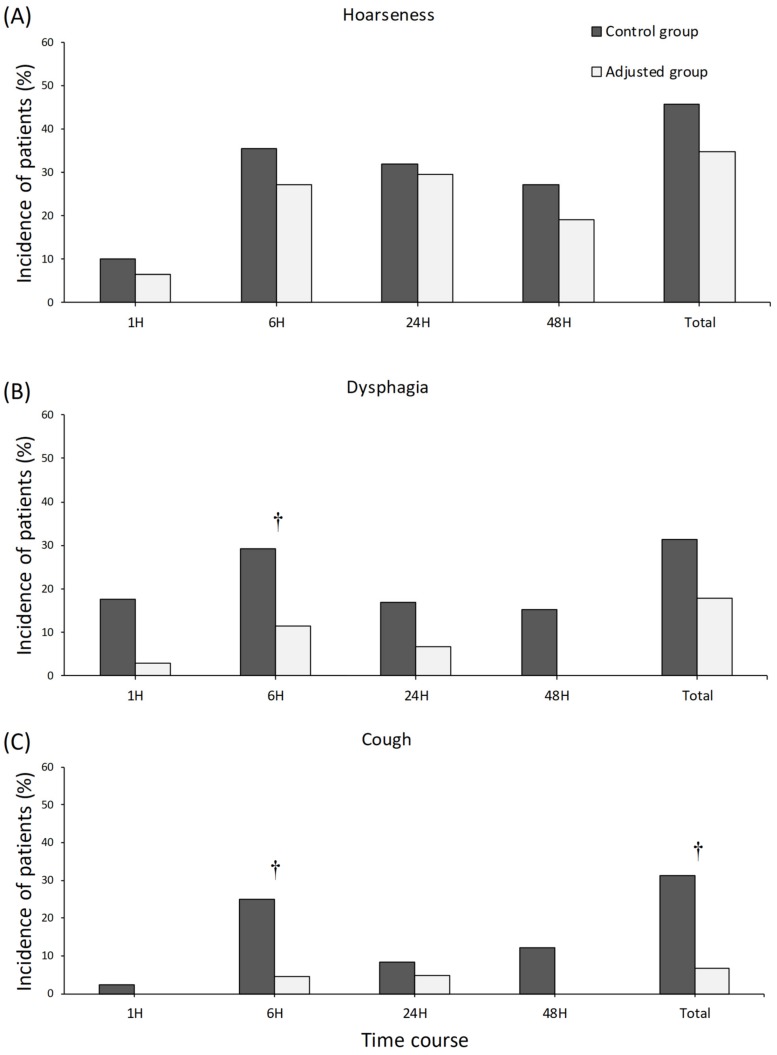
The incidence of laryngo-pharyngeal complications. (**A**) Hoarseness; (**B**) Dysphagia; (**C**) Cough. The incidence of dysphagia and cough 6 h postoperatively was lower in the adjusted group than in the control group (11.4% vs. 29.2%, *p* = 0.035; 4.5% vs. 25%, *p* = 0.006). The total incidence of cough was lower in the adjusted group than in the control group (6.7% vs. 31.3%, *p* = 0.003). † *p* < 0.05.

The total incidences of hoarseness and dysphagia were comparable between the two groups, but there was a significant difference in the incidence of dysphagia at 6 h postoperatively (11.4% for the adjusted group vs. 29.2% for the control group, *p* = 0.035) (Figure 5B). The total incidence of cough during the study period was lower in the adjusted group than in the control group (6.7% vs. 31.3%, *p* = 0.003) (Figure 5C), and there was a significant difference in the incidence of cough at 6 h postoperatively (4.5% for the adjusted group vs. 25% for the control group, *p* = 0.006) (Figure 5C).

### 3.4. Other Recovery Profiles

Patients were evaluated using the modified Aldrete scoring system until ready for discharge from the PACU. The criterion used for patient discharge from the PACU was the achievement of a modified Aldrete score of 9. No difference was observed with respect to PACU recovery time, postoperative pain, and amount of drainage (Table 2).

## 4. Discussion

To the best of our knowledge, this is the first randomized clinical trial evaluating the effect of adjustment of endotracheal cuff pressures on postoperative laryngo-pharyngeal complications after SERT. The results of this present study suggest that the adjustment of endotracheal cuff pressure during surgery decreased the incidence of laryngo-pharyngeal complications, such as POST and cough, after surgery.

Laryngo-pharyngeal complications, such as POST, hoarseness, dysphagia, and cough, may occur after general anesthesia [14], but it is worth nothing that these laryngo-pharyngeal complications develop in most patients after thyroidectomy [7]. Since these complications are mainly associated with endotracheal intubation and cuff pressure, the cuff pressure of the endotracheal tube is monitored continuously in patients with open thyroidectomy [8]. The cuff pressure of the control (unadjusted) group was slightly increased during thyroidectomy, which may be explained by a manipulation of the thyroid that is adjacent to the trachea [8]. The current study evaluated laryngo-pharyngeal complications after SERT, during which CO_2_ gas was inflated to the operative field for surgical condition. The result of the present study showed that the endotracheal cuff pressure was drastically increased during neck extension and decreased right after surgery when the cuff pressure was set at 25 cmH_2_O right after intubation, and then it was left unadjusted during the operation. During SERT, CO_2_ gas was inflated to the operative field with a pressure of 6 mmHg for better surgical conditions, and the endotracheal cuff pressure was maintained around 30 mmHg until the end of the operation. 

The incidence and degree of POST after the adjustment of the endotracheal cuff pressure were compared in patients. The incidence was lower (53.3% vs. 74.5%) and the degree was lesser in patients with adjusted endotracheal cuff pressure than those in patients with unadjusted cuff pressure at postoperative 24 h. The result of this study suggests that the adjustment of endotracheal cuff pressure during thyroidectomy may reduce the incidence and degree of POST. The high prevalence of POST after thyroidectomy may be attributed to the aggravation of the tracheal irritation via airway instrumentation induced by thyroid manipulation during thyroidectomy [8].

The incidences of hoarseness and dysphagia were assessed, and it was found that the adjustment of endotracheal cuff pressure did not reduce the incidence of hoarseness and dysphagia after SERT, except for dysphagia at postoperative 6 h. A vocal cord exam was performed by an otolaryngologist in all patients preoperatively, and patients with free vocal cord movement were enrolled. Additionally, the neural integrity monitor EMG tracheal tubes were used to monitor the recurrent laryngeal nerve during surgery. Postoperative laryngoscopy was performed in patients with transient hoarseness after surgery. A previous investigation with open thyroidectomy reported the incidence in adjusted and unadjusted groups to be 55% [8]. It is interesting to note that the incidence of hoarseness in this present study was 34.8% in the adjusted group and 45.8% in the control (unadjusted) group. Song et al. prospectively evaluated the voice functions and suggested that robotic thyroidectomy has advantages in terms of recovery of voice symptoms over conventional open thyroidectomy, which might be due to reduced adhesion of the laryngo-tracheal structure [15].

It is worth noting that the adjustment of endotracheal cuff pressure reduced the incidence of cough after surgery. Cough after thyroidectomy is a nonspecific reflex that is stimulated by multiple factors; hence, this finding may be due to less irritation of the trachea wall by adjustment of endotracheal tube cuff pressure during thyroid manipulation. 

The current study has a few limitations to be considered. First, layrngo-pharyngeal complications after SERT were evaluated for only two days (48 h) after surgery. Laryngo-pharyngeal complications are known to persist long after thyroidectomy [3]. A further study on the long-term effect of the adjustment of endotracheal cuff pressure during thyroidectomy is needed. Second, the operation was performed by a single experienced surgeon to keep a constant surgical stimulus in this study. SERT required multiple steps and the operator must be familiar with all these steps involved in this new technique. Therefore, the incidence of laryngo-pharyngeal complications may vary depending on the surgeon’s technical skills and knowledge. Third, this study evaluated subjective laryngo-pharyngeal symptoms without objective assessment such as acoustic parameters and aerodynamic study. Cough is a highly subjective symptom that may be influenced by other parameters such as history of smoking. After thyroidectomy, the discordance is found in between the objective parameters and subjective symptoms for approximately one-third of patients who undergo thyroidectomy [16]. Additionally, subjective symptoms without laryngeal nerve injury are found to be more common than objective acoustic parameter abnormalities after thyroidectomy. Finally, this study was performed with the relatively homogenous patient population in the single center, and surgeries were conducted by a single experienced surgeon. Therefore, clinical applications of this study should be interpreted carefully considering various clinical environments such as the patient’s condition or surgeon’s experience. 

## 5. Conclusions

In conclusion, adjustment of endotracheal cuff pressure during SERT reduced the incidence of POST and cough after surgery. Continuous monitoring and adjustment of endotracheal cuff pressure is needed to reduce laryngo-pharyngeal complications after SERT.

## Figures and Tables

**Table 1:** Patients and surgery characteristics.

	Control Group (*n* = 49)	Adjusted Group (*n* = 46)	*p*-Value
Demographics			
Age, mean (SD), y	38.3 (10.3)	38.9 (9.9)	0.787
Female, No. (%)	41 (83.7)	37 (80.4)	0.681
Weight, median (IQR), kg	60.2 (55.45–69.30)	58.75 (54.50–71.75)	0.710
Height, mean (SD), cm	162.7 (7.1)	162.9 (8.2)	0.869
ASA class, No. (%)			0.258
I	47 (95.9)	41 (89.1)	
II	2 (4.1)	5 (10.9)	
Number of intubation, median (IQR)	1 (1–1)	1 (1–1)	1.000
Cormack-Lehane Classification, number (%)			0.110
I	49 (100)	43 (93.5)	
II	0	3 (6.5)	
Operative parameter			0.303
Unilateral lobectomy, No (%)	28 (57.1)	31 (67.4)	
Total thyroidectomy, No (%)	21 (42.9)	15 (32.6)	
Duration, median (IQR), min			
Anesthesia time	175 (160–210)	175 (155–205)	0.641
Operation time	140 (120–165)	135 (115–160)	0.349
Pathologic diagnosis, No (%)			0.379
Papillary thyroid carcinoma	48 (98.0)	44 (95.7)	
Follicular adenoma	0	1 (2.2)	
Medullary carcinoma	1 (2.0)	0	
Diffuse hyperplasia	0	1 (2.2)	
Tumor size, median (IQR), cm	0.9 (0.7–1.2)	1.0 (0.6–1.5)	0.961
Resected thyroid weight, median (IQR), g	10 (7–12)	9.5 (7–12)	0.917

Abbreviations: NC, not calculated; SD, standard deviation; IQR, interquartile range; ASA class, American Society of Anesthesiologist physical class.

**Table 2:** PACU stay, postoperative pain, and amount of drainage.

	Control Group (*n* = 49)	Adjusted Group (*n* = 46)	*p*-Value
PACU stay, median (IQR), min	30 (27–32)	28.5 (25–31)	0.332
Postoperative Pain, median (IQR)			
PO 1h	60 (45–60)	60 (40–70)	0.973
PO 6 h	50 (30–50)	40 (30–60)	0.931
PO 24 h	30 (20–30)	30 (20–30)	0.464
PO 48 h	20 (20–30)	20 (20–30)	0.987
Amount of drainage, median (IQR), mL			
PO 6 h	43 (30–54)	40 (25–58)	0.531
PO 24 h	70 (54.5–81)	72 (48–90)	0.687
PO 48 h	18 (8–35)	15.5 (5–30)	0.985

Abbreviations: PACU, postanesthesia unit. Patients were evaluated using the modified Aldrete scoring system until ready for discharge from the PACU and the criterion used for patient discharge from PACU was the achievement of a modified Aldrete score of 9. Postoperative pain were evaluated using the numerical rating scale (NRS, on a scale from 0, no pain, to 100, most severe pain imaginable) at postoperative at 1, 6, 24, and 48 h after surgery.
